# Supplementary material for: Evolution of anticipatory effects mediated by epigenetic changes
Source: Environ Epigenet. 2022 Apr 22;8(1):dvac007. doi: 10.1093/eep/dvac007 (PMC9031056; doi:10.1093/eep/dvac007)
Supplement: dvac007_Supp [file dvac007_supp.zip › Anticipatory_suppl_revised.pdf]

Evolution of anticipatory effects mediated by epigenetic changes – Supplementary Information

Supplementary Figures

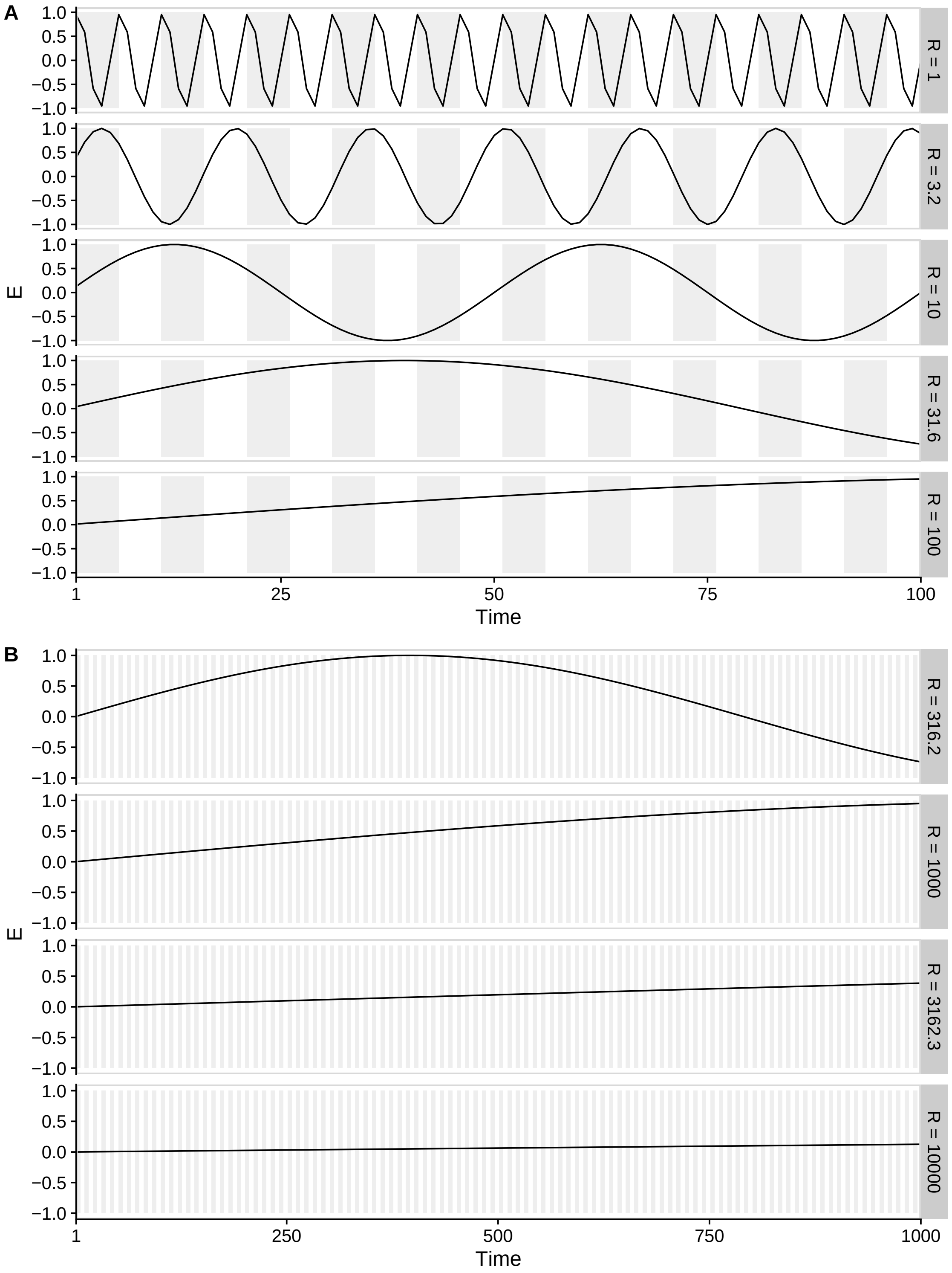

Figure S1: Illustration of how the environment fluctuates with different values of R used in the simulations. To illustrate different rates of environmental change, slower fluctuations are plotted on a different scale. A) Twenty generations are plotted. B) Two hundred generations are plotted. Each generation is five timesteps long, as shown by alternation of shading.

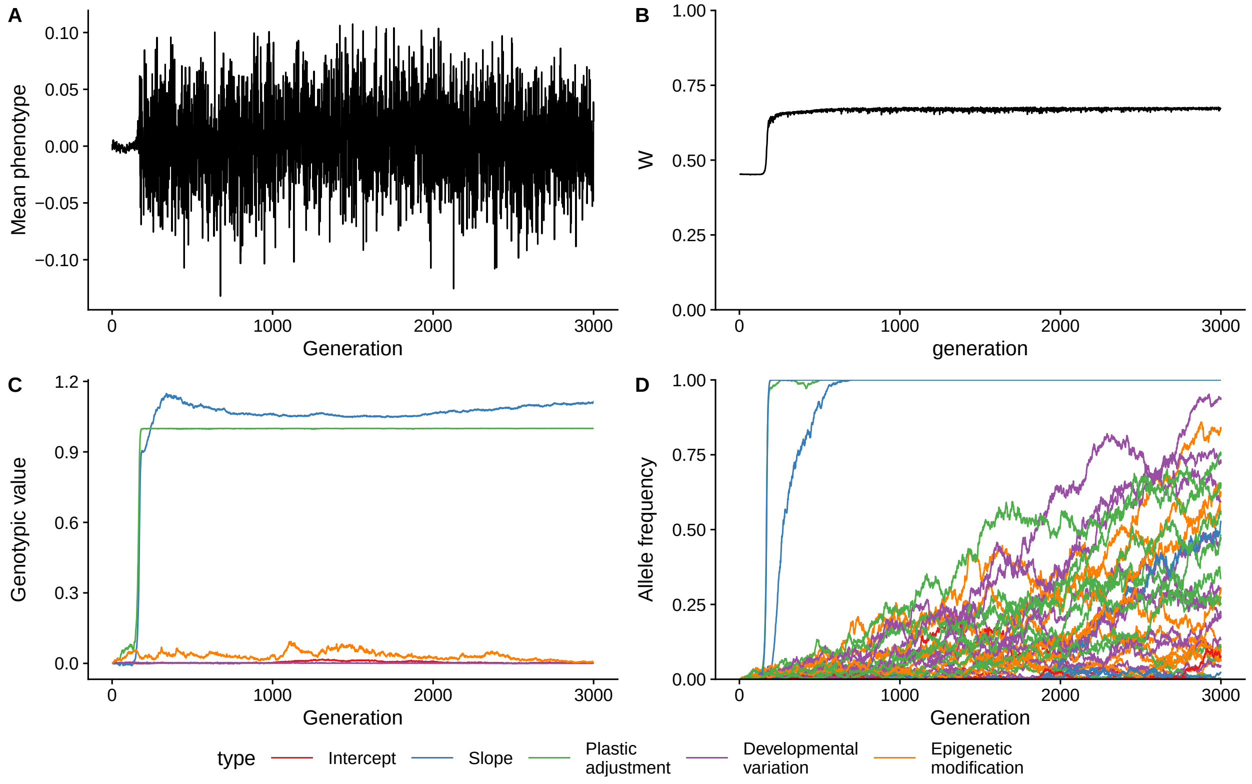

Figure S2: An example of how a single population evolves a strategy of reversible phenotypic plasticity. Simulation parameters were: $R = 1$, $P = 0.9$, $k_d = k_e = 0.02$, and $k_a = 0.01$. A) Phenotypic mean of the population for each generation. B) Population mean fitness for each generation. C) Genotypic values for reaction norm intercept, slope, probability of plastic adjustment, developmental variation, and probability of epigenetic modification for each generation. D) Allele frequencies of derived alleles at QTL controlling the genotypic values. Legend shows colours for types of genotypic values or loci.

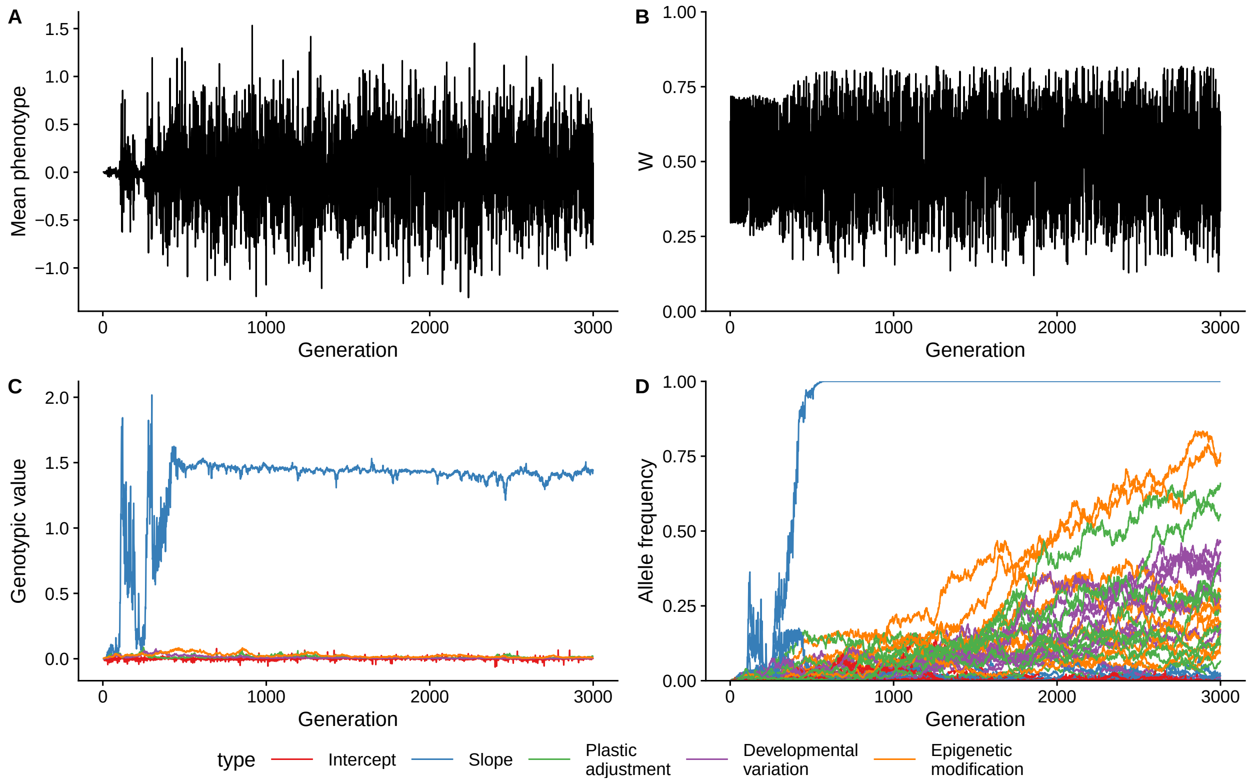

Figure S3: An example of how a single population evolves a strategy of developmental phenotypic plasticity. Simulation parameters were: $R = 10$, $P = 0.2$, $k_d = k_e = 0.02$, and $k_a = 0.01$. A) Phenotypic mean of the population for each generation. B) Population mean fitness for each generation. C) Genotypic values for reaction norm intercept, slope, probability of plastic adjustment, developmental variation, and probability of epigenetic modification for each generation. D) Allele frequencies of derived alleles at QTL controlling the genotypic values.

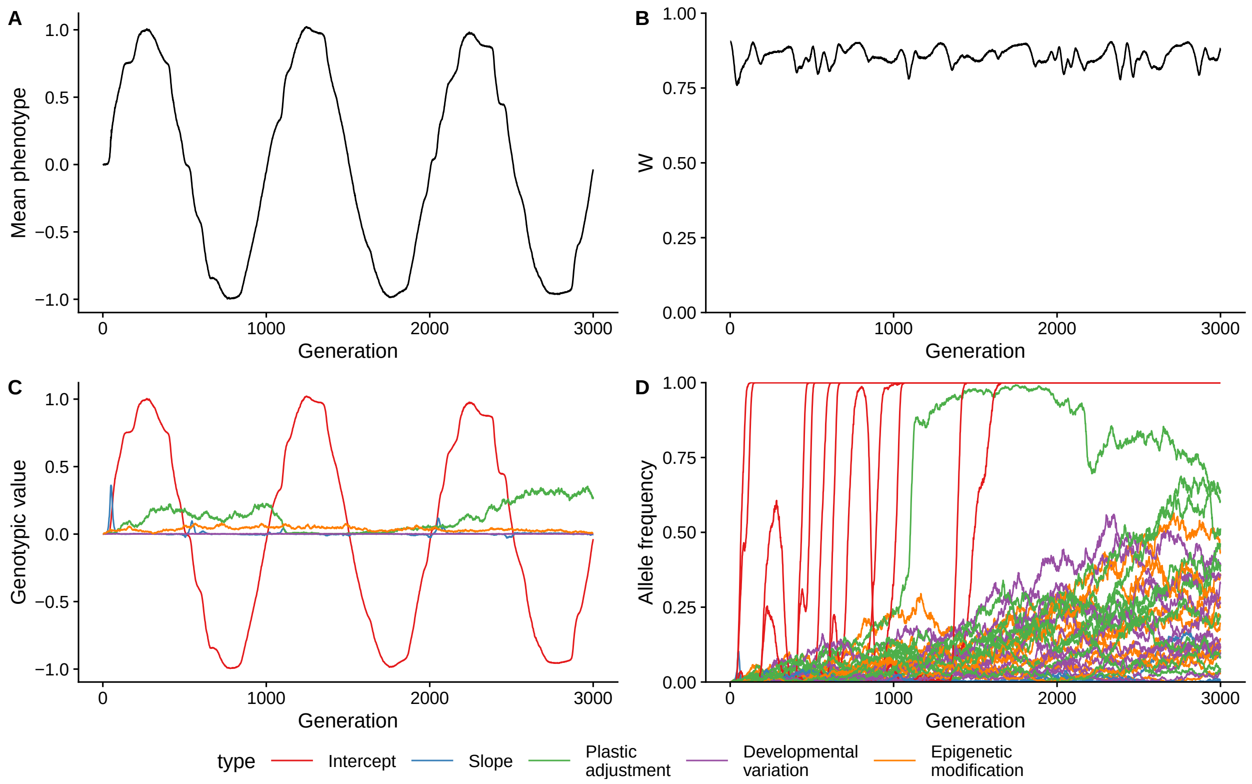

Figure S4: An example of how a single population evolves by tracking the environmental optimum by changing the reaction norm intercept. Simulation parameters were: $R = 1000$, $P = 0.9$, $k_d = k_e = 0.02$, and $k_a = 0.01$. A) Phenotypic mean of the population for each generation. B) Population mean fitness for each generation. C) Genotypic values for reaction norm intercept, slope, probability of plastic adjustment, developmental variation, and probability of epigenetic modification for each generation. D) Allele frequencies of derived alleles at QTL controlling the genotypic values.

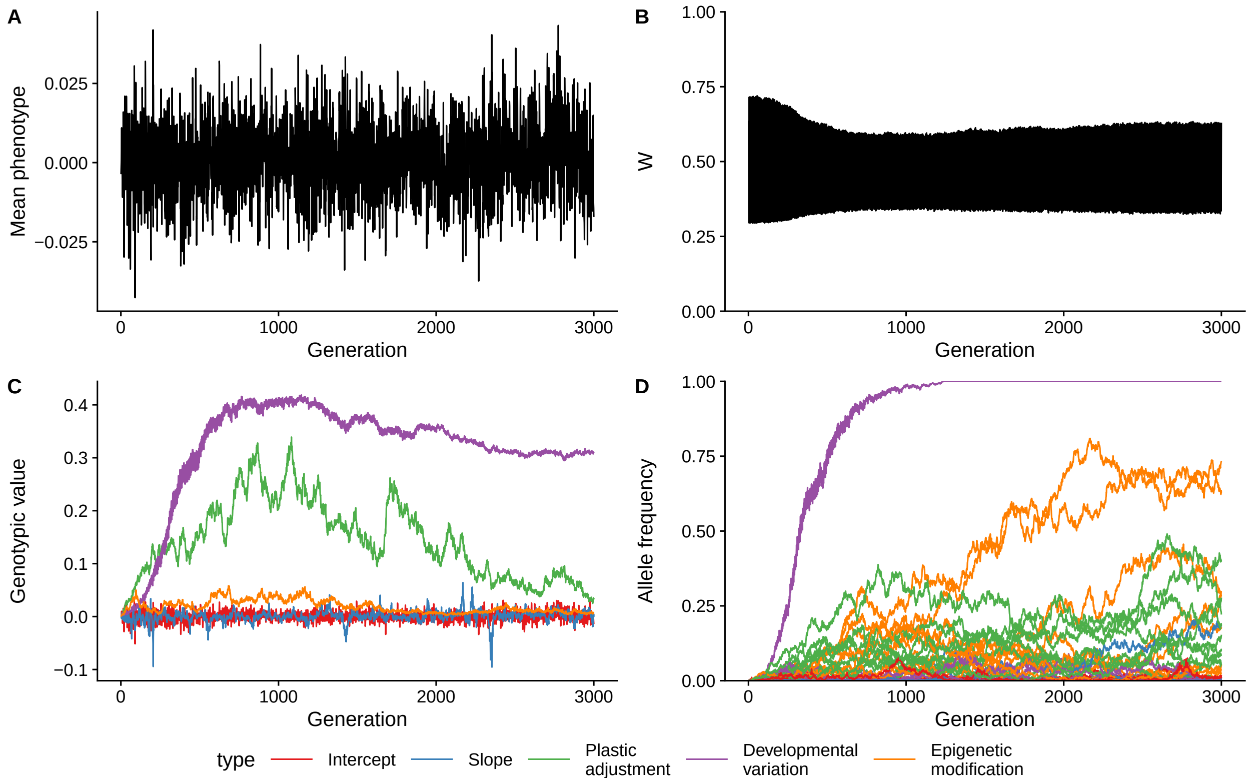

Figure S5: An example of how a single population evolves a strategy of diversifying bet-hedging. Simulation parameters were: $R = 10$, $P = 0$, $k_d = k_e = 0.02$, and $k_a = 0.01$. A) Phenotypic mean of the population for each generation. B) Population mean fitness for each generation. C) Genotypic values for reaction norm intercept, slope, probability of plastic adjustment, developmental variation, and probability of epigenetic modification for each generation. D) Allele frequencies of derived alleles at QTL controlling the genotypic values.

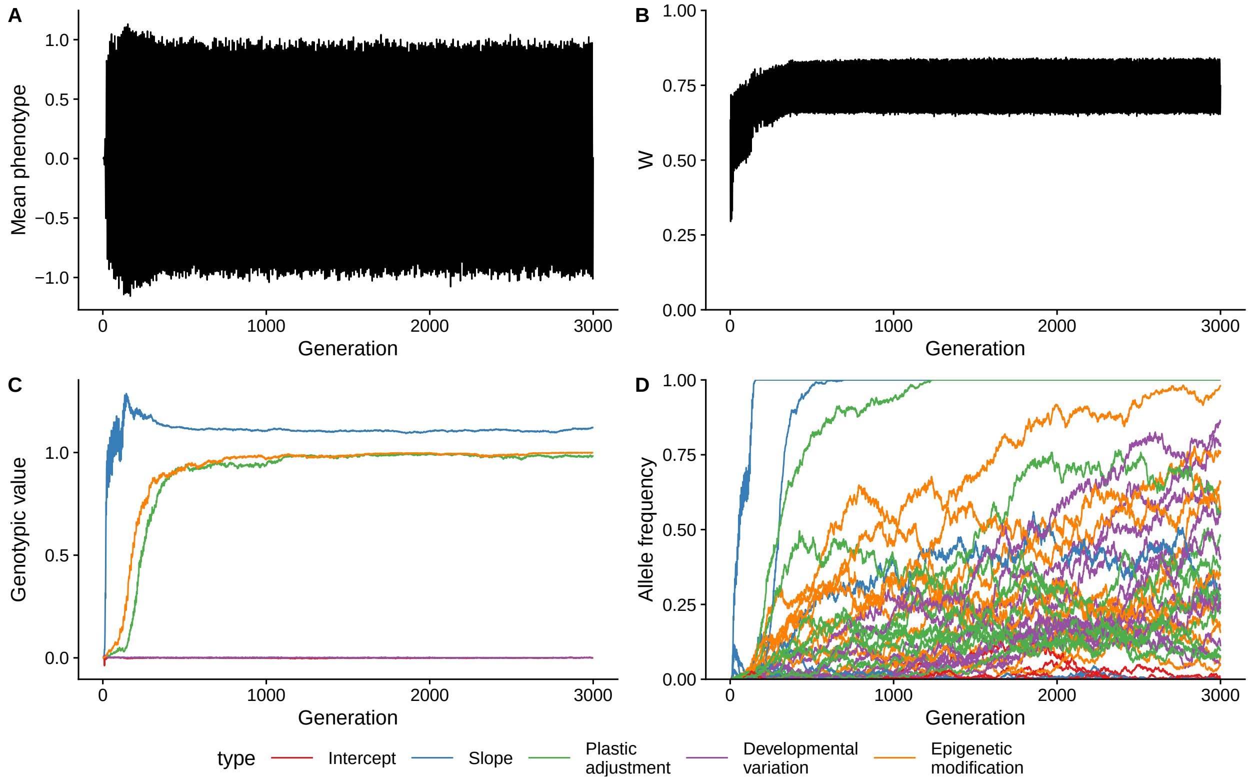

Figure S6: An example of how a single population evolves a strategy of reversible phenotypic plasticity and anticipatory effects via epigenetic modifications. Simulation parameters were: $R = 10$, $P = 0.9$, $k_d = k_e = 0.02$, and $k_a = 0.01$. A) Phenotypic mean of the population for each generation. B) Population mean fitness for each generation. C) Genotypic values for reaction norm intercept, slope, probability of plastic adjustment, developmental variation, and probability of epigenetic modification for each generation. D) Allele frequencies of derived alleles at QTL controlling the genotypic values.

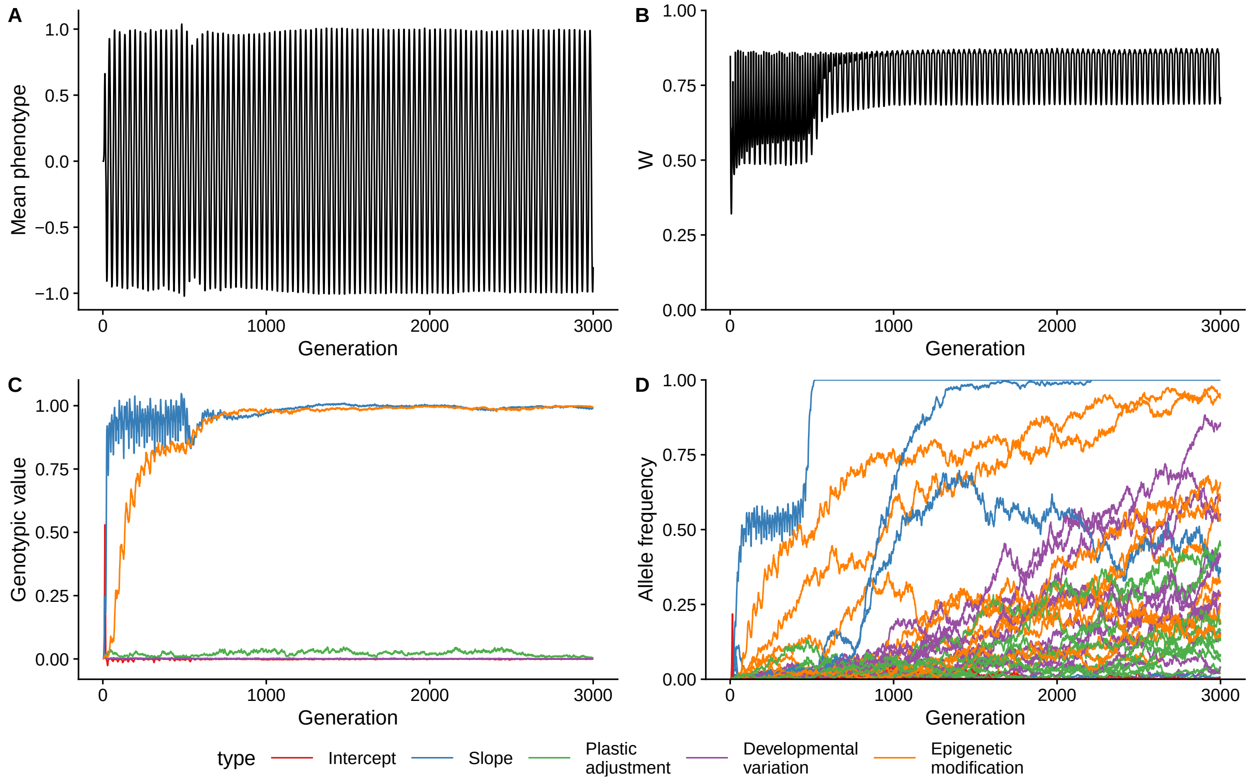

Figure S7: An example of how a single population evolves a strategy of developmental phenotypic plasticity and anticipatory effects via epigenetic modifications. Simulation parameters were: $R = 31.6$, $P = 1$, $k_d = k_e = 0.02$, and $k_a = 0.01$. A) Phenotypic mean of the population for each generation. B) Population mean fitness for each generation. C) Genotypic values for reaction norm intercept, slope, probability of plastic adjustment, developmental variation, and probability of epigenetic modification for each generation. D) Allele frequencies of derived alleles at QTL controlling the genotypic values.

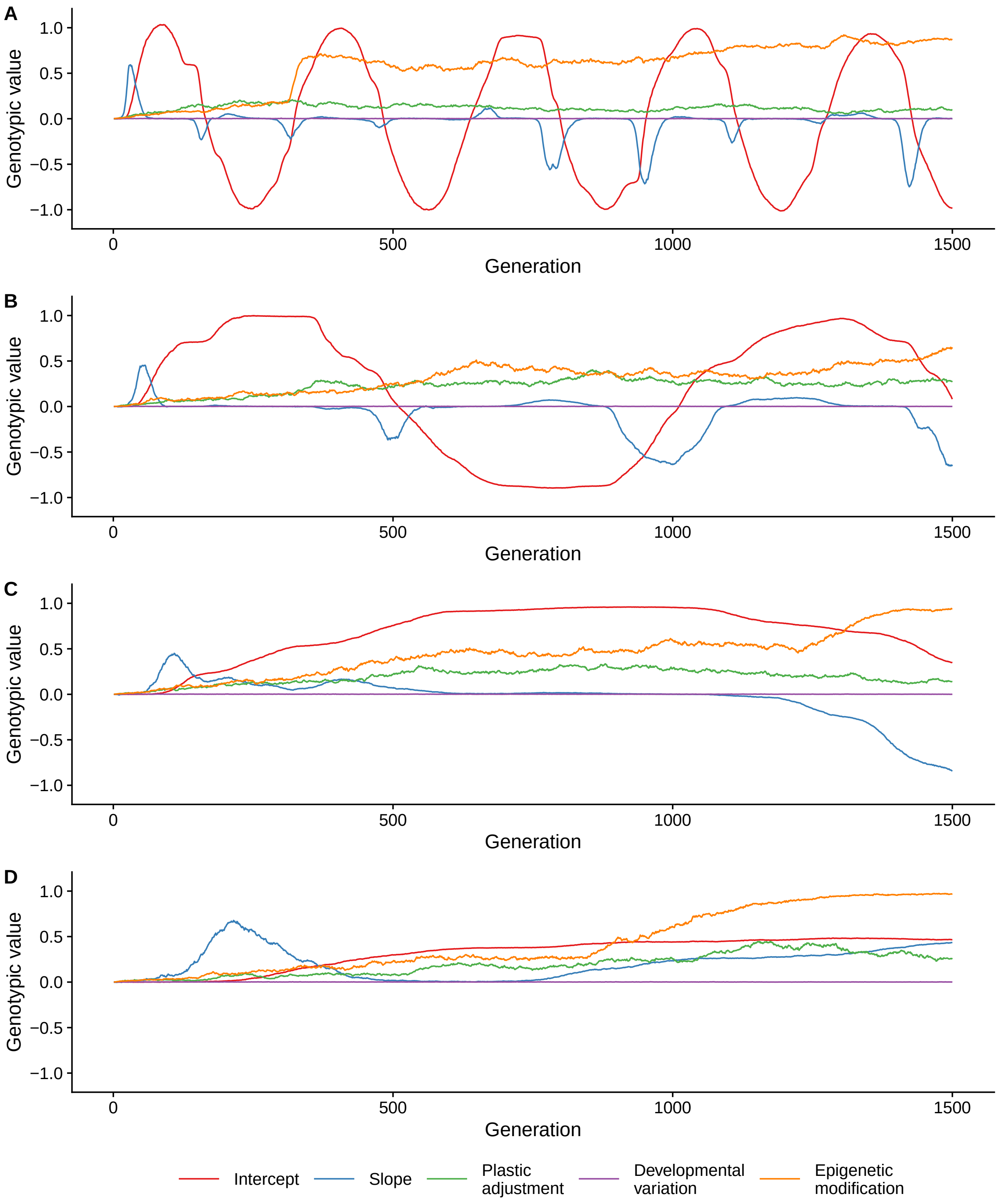

Figure S8: Examples of transient dynamics of plasticity and anticipatory effects punctuated by genetic assimilation. Genotypic values are shown for individual simulations runs. In all panels costs of plasticity are zero, $k_d = k_e = k_a = 0$, and $P = 1$. A) $R = 316.2$ B) $R = 1000$ C) $R = 3162.3$ D) $R = 10000$

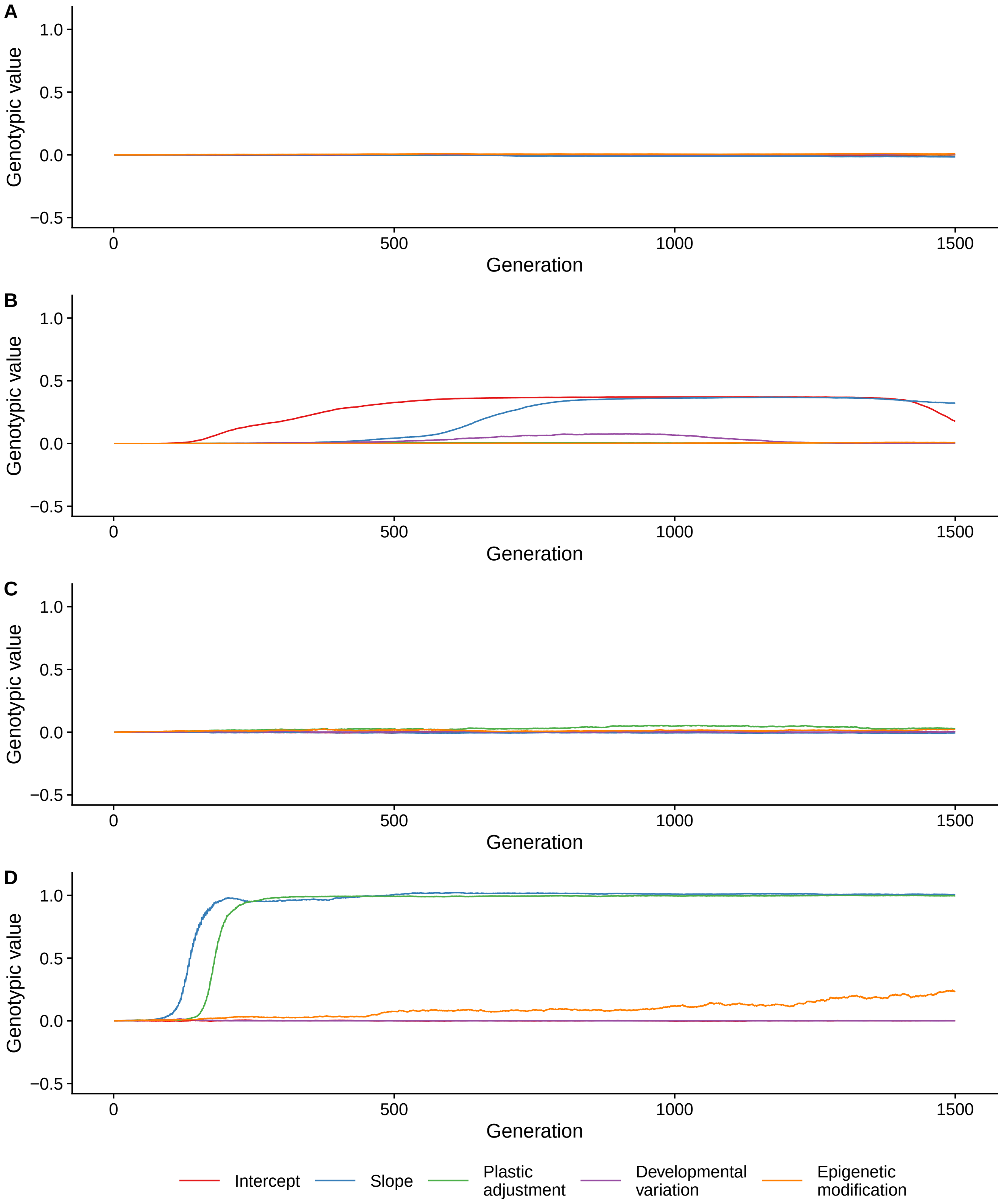

Figure S9: Examples of evolutionary trajectories when standard deviation of mutational effects is low. Genotypic values are shown for individual simulation runs. In all panels number of loci is 10 for each category, $P = 1$, $k_d = k_e = 0.02$, and $k_a = 0.01$. A) $R = 1$ ($\log_{10} R = 0$), $\sigma_\alpha = 0.01$ B) $R = 3162.3$ ($\log_{10} R = 3.5$), $\sigma_\alpha = 0.01$ C) $R = 1$ ($\log_{10} R = 0$), $\sigma_\alpha = 0.1$ D) $R = 3.2$ ($\log_{10} R = 0.5$), $\sigma_\alpha = 0.1$
